# Supplementary material for: A Likelihood Approach for Real-Time Calibration of Stochastic Compartmental Epidemic Models
Source: PLoS Comput Biol. 2017 Jan 17;13(1):e1005257. doi: 10.1371/journal.pcbi.1005257 (PMC5240920; doi:10.1371/journal.pcbi.1005257)
Supplement: S1 File — (TAR.GZ) [file pcbi.1005257.s014.tar.gz › HSPH_Online-SI-Revision/EnKF07/n10-mild/n10-mild_EnKF_table-4.pdf]

1	R0	{1.25046, 6.80278}	{2.7702, 11.3766}	{{2.32684, 3.7293}, {3., 16.3158}}	{121.534, 100.57}	inf	157.	674.565	{36.2885, 633.573}	361.084	0.617915	newly	177.	451.97	{30., 772.}	191.938	0.456236	Reff	1.16118	0.542136	{0.256362, 0.842411}	61.1635	ar	3516.	17 404.9	{15 617., 21 452.}	395.018
2	R0	{1.32013, 4.45847}	{2.41148, 5.02087}	{{1.47726, 2.39598}, {3., 9.88483}}	{82.6702, 71.7317}	inf	184.	273.606	{34.0312, 779.044}	76.5882	0.269779	newly	307.	648.35	{22., 2383.}	177.762	0.459469	Reff	1.21267	0.59763	{0.243722, 1.4635}	57.4633	ar	4420.	18 360.8	{16 971., 23 668.}	315.403
3	R0	{1.31785, 5.36796}	{2.13888, 3.91223}	{{0.932781, 2.68688}, {3., 5.26216}}	{76.1139, 35.1379}	inf	142.	1299.13	{345.171, 2606.38}	816.553	0.835062	newly	232.	1458.48	{391., 4146.}	530.534	0.662807	Reff	1.23206	0.377026	{0.115758, 0.792402}	69.3987	ar	4942.	14 460.5	{7515., 18 920.}	192.917
4	R0	{1.38801, 6.16031}	{2.4439, 6.83324}	{{0.918666, 1.51892}, {3., 19.5575}}	{88.0912, 109.617}	inf	202.	368.306	{43.3907, 421.49}	129.037	0.353958	newly	273.	930.21	{40., 1109.}	322.839	0.541623	Reff	1.26267	0.586446	{0.206578, 0.824869}	64.393	ar	4790.	18 135.5	{16 643., 18 614.}	278.611
5	R0	{1.27955, 11.57}	{2.82674, 8.38176}	{{2.25304, 3.79924}, {3., 13.5994}}	{120.917, 39.6203}	inf	185.	915.737	{66.0915, 2232.19}	424.214	0.450116	newly	121.	1054.53	{46., 4031.}	813.479	0.59947	Reff	1.13842	0.583897	{0.231249, 1.46658}	68.8643	ar	3460.	18 933.7	{16 388., 25 532.}	447.215
6	R0	{1.22184, 4.34395}	{2.5243, 5.6027}	{{1.81826, 0.905669}, {3., 13.2013}}	{107.751, 84.797}	inf	108.	2371.62	{523.507, 5496.86}	2096.49	1.21391	newly	215.	2620.83	{540., 6710.}	1120.67	0.926499	Reff	1.17138	0.624832	{0.211002, 1.33926}	53.5021	ar	3524.	15 179.9	{9951., 24 493.}	330.757
7	R0	{1.23856, 4.59068}	{2.42856, 5.28682}	{{1.47642, 2.39919}, {3., 10.0339}}	{96.0793, 73.6723}	inf	160.	348.129	{34.7782, 1021.83}	140.641	0.320239	newly	265.	720.15	{22., 3254.}	230.215	0.458914	Reff	1.13155	0.583296	{0.110201, 1.45595}	57.2158	ar	3855.	18 519.8	{16 910., 24 977.}	380.411
8	R0	{1.34531, 10.6689}	{2.73867, 9.54232}	{{2.32015, 3.78978}, {3., 14.0788}}	{103.571, 46.0826}	inf	312.	654.871	{40.5785, 1060.63}	171.234	0.524558	newly	198.	524.96	{15., 689.}	216.131	0.562806	Reff	1.20365	0.580543	{0.276474, 0.926083}	56.6297	ar	4852.	17 482.7	{15 585., 23 366.}	260.319
9	R0	{1.31775, 5.19226}	{2.13478, 9.44587}	{{0.460443, 5.80518}, {3., 14.4194}}	{96.4653, 81.9221}	inf	120.	443.975	{54.7845, 308.574}	290.678	0.504535	newly	202.	340.59	{19., 320.}	116.361	0.367943	Reff	1.22551	0.385463	{0.0722944, 0.673316}	68.5466	ar	4708.	17 845.6	{16 883., 18 443.}	279.048
10	R0	{1.25916, 6.89207}	{2.44821, 6.1097}	{{0.688249, 1.86681}, {3., 9.24349}}	{112.516, 46.0235}	inf	175.	702.123	{38.4198, 1687.5}	351.678	0.479519	newly	191.	863.62	{24., 4479.}	407.058	0.565069	Reff	1.10151	0.639971	{0.118272, 0.919453}	58.6302	ar	3823.	18 108.9	{16 026., 23 919.}	373.683
11	R0	{1.20377, 5.46566}	{2.50743, 5.74364}	{{2.01554, 4.05337}, {3., 7.92994}}	{109.418, 32.1502}	inf	147.	414.298	{16.2782, 546.214}	222.733	0.377741	newly	200.	438.47	{11., 1100.}	184.695	0.472169	Reff	1.11577	0.544079	{0.164536, 1.17799}	64.8758	ar	3114.	18 031.1	{16 596., 21 782.}	479.533
12	R0	{1.27878, 2.55804}	{2.01655, 4.34385}	{{1.15075, 2.67918}, {3., 5.15547}}	{69.5502, 69.8116}	inf	57.	1486.78	{337.564, 5165.41}	2509.54	1.25645	newly	277.	1624.3	{446., 5374.}	491.025	0.638466	Reff	1.24259	0.412513	{0.119749, 0.907338}	67.5242	ar	3930.	15 497.6	{6131., 26 047.}	296.597
13	R0	{1.41227, 5.45696}	{2.75968, 9.14964}	{{2.32166, 3.81219}, {3., 14.0904}}	{95.4075, 96.4172}	inf	188.	675.276	{29.329, 1221.35}	309.371	0.551892	newly	295.	528.32	{20., 703.}	145.41	0.509499	Reff	1.28587	0.673393	{0.236316, 1.27521}	56.9152	ar	4822.	17 776.5	{15 482., 23 443.}	268.655
14	R0	{1.32917, 7.27593}	{2.37043, 8.63685}	{{0.715476, 0.00818666}, {3., 11.9784}}	{101.278, 52.1468}	inf	250.	827.07	{17.0205, 1951.01}	293.007	0.609466	newly	248.	746.56	{9., 1887.}	272.5	0.697008	Reff	1.18482	0.556588	{0.0976167, 0.826735}	61.2285	ar	4758.	18 062.	{15 900., 21 310.}	279.613
15	R0	{1.31629, 9.97481}	{2.45757, 10.0397}	{{1.07859, 1.75834}, {3., 19.6872}}	{94.766, 87.3228}	inf	186.	357.95	{24.0691, 484.222}	143.528	0.407592	newly	149.	738.23	{26., 699.}	477.188	0.600583	Reff	1.22178	0.604005	{0.191499, 0.888754}	63.771	ar	4543.	18 215.8	{16 606., 20 541.}	300.964
16	R0	{1.36735, 5.74166}	{2.21667, 8.97962}	{{0.464159, 5.79867}, {3., 14.7578}}	{92.1308, 56.3941}	inf	182.	325.188	{52.1293, 306.08}	102.884	0.341139	newly	266.	258.69	{23., 302.}	52.703	0.349597	Reff	1.26507	0.399538	{0.0837865, 0.685248}	69.2281	ar	4516.	17 749.5	{16 951., 18 194.}	293.035
17	R0	{1.27524, 13.2396}	{2.38269, 10.1063}	{{0.732365, 6.84139}, {3., 11.656}}	{107.194, 23.6662}	inf	80.	875.345	{7.06784, 3637.32}	1024.72	0.841878	newly	45.	594.58	{4., 2493.}	1250.93	0.913016	Reff	1.24591	0.687536	{0.139726, 1.46344}	60.5242	ar	3731.	18 247.1	{15 842., 25 683.}	389.067
18	R0	{1.21884, 7.71545}	{2.43443, 7.3302}	{{0.713372, 0.0506468}, {3., 11.2604}}	{117.228, 64.4939}	inf	167.	913.913	{12.9431, 3293.45}	486.79	0.657556	newly	159.	934.61	{7., 2511.}	528.736	0.752532	Reff	1.1234	0.654739	{0.105817, 0.86928}	59.5405	ar	3080.	18 330.8	{15 943., 25 386.}	495.155
19	R0	{1.34879, 6.71464}	{2.08376, 9.14104}	{{0.416686, 5.70133}, {3., 14.4247}}	{91.2428, 54.2452}	inf	339.	395.751	{41.6688, 305.962}	72.211	0.346038	newly	410.	311.74	{18., 384.}	82.0195	0.585672	Reff	1.15092	0.399904	{0.0533185, 0.666282}	65.9818	ar	4585.	17 931.5	{16 979., 20 962.}	291.091
20	R0	{1.37284, 5.75036}	{2.45413, 5.20502}	{{1.48995, 2.40773}, {3., 10.0111}}	{78.7626, 63.2957}	inf	216.	333.663	{30.7898, 1037.24}	97.8975	0.326793	newly	303.	721.07	{20., 3317.}	204.155	0.487464	Reff	1.23597	0.593236	{0.213424, 1.47145}	57.6971	ar	4750.	18 402.6	{16 897., 24 959.}	287.422
21	R0	{1.29938, 5.36419}	{2.45148, 5.23123}	{{1.47704, 2.39823}, {3., 9.7121}}	{88.665, 62.9553}	inf	198.	355.478	{24.7405, 1074.39}	119.036	0.30852	newly	270.	702.36	{13., 4111.}	219.941	0.470626	Reff	1.18387	0.580719	{0.0544745, 1.46073}	59.402	ar	4337.	18 515.7	{16 802., 25 213.}	326.925
22	R0	{1.31882, 7.47626}	{2.35151, 9.74355}	{{0.699424, 6.85623}, {3., 11.8495}}	{102.575, 37.293}	inf	227.	875.199	{15.3877, 1928.97}	344.6	0.66044	newly	235.	609.46	{7., 1004.}	228.119	0.670794	Reff	1.193	0.559885	{0.0968481, 0.855}	61.5273	ar	3977.	18 167.5	{15 856., 21 512.}	356.814
23	R0	{1.26215, 3.46375}	{2.47514, 5.72395}	{{1.2468, 4.18452}, {3., 7.74314}}	{99.0629, 65.2529}	inf	117.	264.816	{11.5479, 267.03}	158.09	0.28944	newly	283.	270.64	{9., 411.}	110.332	0.609829	Reff	1.18314	0.419132	{0.157253, 1.04501}	69.9143	ar	3607.	17 624.5	{16 529., 18 990.}	388.619
24	R0	{1.16712, 4.19464}	{2.60873, 6.89085}	{{1.67521, 3.52621}, {3., 11.0864}}	{123.518, 79.2568}	inf	122.	516.242	{29.482, 401.719}	356.455	0.536158	newly	227.	353.34	{22., 592.}	105.894	0.392355	Reff	1.04609	0.5698	{0.115568, 0.78609}	58.8741	ar	3000.	18 192.7	{16 436., 23 909.}	506.424
25	R0	{1.22751, 3.34517}	{2.39233, 5.09049}	{{1.77883, 0.949345}, {3., 13.1799}}	{97.6756, 102.892}	inf	50.	2425.99	{509.904, 5483.46}	4752.55	1.52898	newly	113.	3005.92	{502., 7987.}	2561.31	1.20149	Reff	1.20861	0.619187	{0.165986, 1.34296}	54.7644	ar	3086.	15 059.8	{7170., 24 709.}	388.293
26	R0	{1.18747, 5.88083}	{2.7358, 12.8037}	{{2.31038, 6.76573}, {3., 16.2245}}	{130.389, 117.718}	inf	178.	540.846	{135.089, 673.29}	210.771	0.309644	newly	234.	270.69	{58., 360.}	75.8504	0.277949	Reff	1.02301	0.601013	{0.270793, 1.09953}	52.3812	ar	3614.	17 246.9	{15 634., 20 825.}	377.224
27	R0	{1.29023, 4.76202}	{2.53387, 6.86065}	{{1.68256, 3.51046}, {3., 9.18874}}	{96.3894, 65.6214}	inf	206.	496.567	{123.372, 1399.98}	160.107	0.292561	newly	330.	514.54	{121., 813.}	135.4	0.419221	Reff	1.17217	0.563129	{0.108134, 0.800084}	63.4878	ar	4245.	18 235.7	{16 388., 26 450.}	329.581
28	R0	{1.2258, 12.9056}	{2.74833, 9.8533}	{{2.18818, 3.46175}, {3., 19.9377}}	{124.207, 55.1295}	inf	188.	461.166	{66.0991, 1167.38}	172.711	0.400572	newly	113.	346.08	{61., 888.}	230.088	0.402644	Reff	1.0369	0.538521	{0.0646237, 1.14034}	66.6505	ar	3639.	14 559.4	{12 272., 19 430.}	300.094
29	R0	{1.34702, 6.29049}	{2.48869, 8.51464}	{{1.01128, 1.65782}, {3., 19.4269}}	{93.2507, 123.193}	inf	264.	393.615	{60.1141, 795.674}	105.665	0.363225	newly	312.	969.65	{42., 1915.}	305.029	0.630441	Reff	1.19265	0.613513	{0.235203, 0.746227}	62.3109	ar	4560.	18 182.8	{16 484., 19 442.}	298.746
30	R0	{1.37931, 6.78741}	{2.53783, 5.032}	{{1.68148, 0.334954}, {3., 7.19068}}	{83.9929, 32.3983}	inf	251.	472.007	{207.467, 588.057}	117.228	0.200469	newly	287.	651.22	{140., 667.}	177.275	0.285425	Reff	1.17614	0.581042	{0.155816, 0.801825}	62.1972	ar	4667.	18 258.4	{16 456., 25 408.}	291.222
31	R0	{1.16062, 4.47529}	{2.35484, 5.09845}	{{1.70419, 0.852126}, {3., 13.1369}}	{102.895, 78.0731}	inf	80.	2945.18	{511.039, 6190.23}	3581.48	1.40685	newly	156.	3197.82	{521., 7537.}	1949.88	1.14057	Reff	1.12661	0.622114	{0.223225, 1.34541}	54.1016	ar	3037.	16 595.2	{10 784., 25 489.}	446.435
32	R0	{1.37647, 18.7122}	{2.76063, 3.62885}	{{2.10441, 3.42967}, {3., 3.4899}}	{100.559, 80.7416}	inf	360.	245.851	{181.699, 181.699}	67.2051	0.355734	newly	137.	353.27	{167., 262.}	164.912	0.263113	Reff	1.09897	0.466355	{0.161066, 0.616928}	66.0743	ar	4824.	16 483.4	{15 589., 17 577.}	241.695
33	R0	{1.35084, 6.08841}	{2.53851, 7.86974}	{{1.06405, 1.55078}, {3., 19.6443}}	{94.8427, 123.442}	inf	217.	392.917	{53.5427, 845.321}	128.761	0.34034	newly	297.	1093.23	{41., 2575.}	356.987	0.603906	Reff	1.23859	0.613557	{0.234693, 0.880474}	63.1927	ar	4759.	18 332.8	{16 584., 21 787.}	285.224
34	R0	{1.36912, 6.316}	{2.4608, 8.85351}	{{1.0549, 1.72178}, {3., 19.6402}}	{88.905, 124.106}	inf	198.	469.948	{49.4752, 1149.46}	176.101	0.366395	newly	262.	1076.72	{43., 2382.}	394.908	0.592193	Reff	1.2459	0.657217	{0.218265, 0.896072}	64.0478	ar	4537.	18 540.8	{16 598., 22 632.}	308.657
35	R0	{1.35831, 7.09038}	{2.52983, 9.40427}	{{1.14953, 1.66568}, {3., 18.8631}}	{92.8567, 115.069}	inf	331.	342.151	{55.1326, 547.717}	78.5664	0.398033	newly	339.	781.64	{47., 927.}	233.617	0.630603	Reff	1.17304	0.664899	{0.24788, 1.07905}	58.4201	ar	4667.	18 181.9	{16 558., 21 678.}	289.584
36	R0	{1.28315, 5.89938}	{2.46667, 5.5395}	{{1.46752, 2.33202}, {3., 10.003}}	{92.2358, 63.0879}	inf	255.	319.653	{37.2639, 799.393}	83.0486	0.323718	newly	336.	583.16	{75., 1254.}	150.923	0.473447	Reff	1.15291	0.552874	{0.0509448, 1.35967}	58.6921	ar	4460.	18 256.3	{16 916., 22 826.}	309.335
37	R0	{1.19303, 2.75113}	{2.13754, 3.99665}	{{0.880469, 2.6815}, {3., 5.11239}}	{87.2128, 46.4373}	inf	55.	1361.02	{305.211, 2253.}	2375.83	1.2301	newly	184.	1433.67	{373., 4115.}	682.723	0.761938	Reff	1.13565	0.363504	{0.107736, 0.716123}	68.4433	ar	3142.	14 707.3	{6736., 19 120.}	369.803
38	R0	{1.36033, 7.8088}	{2.20939, 8.79672}	{{0.452562, 5.72497}, {3., 14.3454}}	{92.6842, 46.8089}	inf	249.	323.815	{41.1015, 305.942}	61.156	0.301334	newly	256.	263.15	{21., 311.}	57.3242	0.392553	Reff	1.22334	0.41441	{0.0840841, 0.669899}	67.0207	ar	4941.	17 741.4	{16 933., 18 089.}	259.064
39	R0	{1.27353, 3.70845}	{2.42566, 5.26993}	{{1.47538, 2.35963}, {3., 10.0205}}	{90.4676, 86.1619}	inf	114.	326.852	{177.149, 702.021}	200.911	0.382583	newly	257.	586.81	{93., 1225.}	180.673	0.370065	Reff	1.17827	0.611278	{0.247887, 1.34324}	54.2884	ar	3725.	18 287.2	{16 992., 21 244.}	390.93
40	R0	{1.19983, 12.1486}	{2.86367, 3.88323}	{{2.45466, 3.58351}, {3., 4.53083}}	{138.673, 68.0356}	inf	191.	1118.8	{58.9785, 2003.88}	516.903	0.641589	newly	121.	1509.93	{67., 5389.}	1167.2	0.801697	Reff	1.02525	0.513188	{0.201517, 0.531735}	75.7464	ar	3619.	18 404.4	{15 917., 26 838.}	408.55
41	R0	{1.20935, 2.67169}	{2.43724, 5.06586}	{{1.76066, 0.856654}, {3., 13.162}}	{101.533, 128.81}	inf	53.	2398.29	{506.879, 5502.76}	4425.49	1.50057	newly	165.	2671.33	{514., 7210.}	1520.19	1.03575	Reff	1.16702	0.598245	{0.189149, 1.33712}	57.7239	ar	3429.	14 788.5	{8072., 24 711.}	331.276
42	R0	{1.23313, 4.74781}	{2.21315, 4.16449}	{{0.71736, 1.18618}, {3., 5.9691}}	{105.979, 41.9904}	inf	138.	686.216	{261.107, 1120.5}	400.45	0.430026	newly	211.	856.33	{199., 2043.}	310.649	0.324607	Reff	1.10217	0.506306	{0.0971963, 0.805319}	61.7471	ar	3835.	17 960.2	{16 211., 20 166.}	368.323
43	R0	{1.26105, 2.39713}	{2.06201, 3.98654}	{{0.145598, 0.89192}, {3., 5.97611}}	{109.302, 111.561}	inf	88.	446.514	{87.6785, 929.05}	411.602	0.56734	newly	321.	493.8	{72., 1095.}	107.128	0.38241	Reff	1.20809	0.268149	{0.0207756, 0.459024}	77.8038	ar	3723.	16 132.8	{14 488., 18 254.}	334.001
44	R0	{1.36948, 8.88006}	{2.52312, 6.98513}	{{1.04495, 1.52538}, {3., 19.4643}}	{92.6422, 89.4957}	inf	274.	354.212	{48.3084, 484.158}	90.085	0.335246	newly	232.	907.82	{41., 2424.}	367.991	0.531978	Reff	1.23623	0.563082	{0.220507, 0.773615}	62.9159	ar	4437.	17 951.1	{16 558., 20 044.}	304.576
45	R0	{1.34882, 3.3791}	{2.51399, 7.14297}	{{2.12708, 3.91103}, {3., 14.7084}}	{88.4589, 113.203}	inf	49.	1989.39	{334.828, 3834.87}	3962.09	1.45445	newly	143.	2241.56	{301., 5798.}	1472.29	1.01122	Reff	1.32279	0.943036	{0.342693, 2.06222}	48.9608	ar	4422.	14 428.7	{8257., 21 377.}	226.803
46	R0	{1.23774, 4.34917}	{2.01911, 4.08457}	{{0.150574, 0.84883}, {3., 5.99867}}	{105.19, 67.2217}	inf	107.	578.918	{111.977, 1326.97}	446.721	0.569317	newly	199.	645.91	{93., 1296.}	253.613	0.463649	Reff	1.16533	0.292919	{0.0233054, 0.460736}	75.0701	ar	3685.	16 647.4	{14 496., 18 800.}	351.761
47	R0	{1.34327, 4.78811}	{2.14745, 3.64882}	{{0.164759, 0.868326}, {3., 5.99358}}	{100.173, 58.014}	inf	102.	466.934	{110.896, 723.703}	360.77	0.538152	newly	151.	560.69	{98., 1074.}	287.477	0.489312	Reff	1.29693	0.306388	{0.0266012, 0.459799}	76.3758	ar	4517.	16 551.8	{15 317., 18 111.}	266.433
48	R0	{1.24366, 4.25814}	{2.15369, 8.97916}	{{0.464344, 5.78421}, {3., 14.5224}}	{106.354, 110.87}	inf	131.	417.89	{53.4954, 306.854}	238.804	0.458117	newly	260.	316.08	{23., 322.}	74.7308	0.325256	Reff	1.16755	0.362107	{0.0469877, 0.667619}	69.0916	ar	3678.	17 730.5	{16 947., 18 014.}	382.07
49	R0	{1.36017, 7.5415}	{2.7229, 12.378}	{{2.31814, 6.6746}, {3., 15.0866}}	{100.188, 71.4885}	inf	127.	435.076	{92.8946, 643.54}	251.468	0.40517	newly	116.	233.82	{40., 286.}	128.638	0.294645	Reff	1.22946	0.565242	{0.358831, 0.833662}	58.0851	ar	4178.	16 867.2	{15 664., 17 729.}	303.714
50	R0	{1.25333, 6.63578}	{2.71546, 9.80007}	{{2.31262, 3.88829}, {3., 13.0054}}	{116.66, 63.4133}	inf	201.	353.236	{22.193, 467.522}	118.102	0.370059	newly	235.	227.03	{18., 302.}	76.2936	0.413708	Reff	1.0642	0.561291	{0.317953, 0.819073}	55.7856	ar	3401.	16 956.6	{15 636., 17 755.}	398.577
